# Residual mesorectum on postoperative magnetic resonance imaging following transanal total mesorectal excision (TaTME) and laparoscopic total mesorectal excision (LapTME) in rectal cancer

**DOI:** 10.1007/s00464-018-6279-9

**Published:** 2018-07-02

**Authors:** M. Veltcamp Helbach, T. W. A. Koedam, J. J. Knol, A. Diederik, G. J. Spaargaren, H. J. Bonjer, J. B. Tuynman, C. Sietses

**Affiliations:** 1 Department of Surgery, VU University Medical Centre, De Boelelaan 1117, 1081 HV Amsterdam, The Netherlands; 2 Department of Surgery, Gelderse Vallei Hospital, Ede, The Netherlands; 3 Department of Surgery, Jessa Hospital, Hasselt, Belgium

**Keywords:** Rectum, Cancer, TaTME, Mesorectal excision, MRI

## Abstract

**Background:**

The standard treatment for mid- and low-rectal cancer is total mesorectal excision. Incomplete excision is an important predictor of local recurrence after rectal cancer surgery. Transanal TME (TaTME) is a new treatment option in which the rectum is approached with both laparoscopic and transanal endoscopic techniques. The aim of the present study was to determine the prevalence and localisation of residual mesorectal tissue by postoperative magnetic resonance imaging (MRI) of the pelvis and compare this between TaTME and laparoscopic TME (LapTME) patients. In addition, we assessed correspondence with histopathological quality.

**Methods:**

Two groups of patients with cT1–T3 rectal cancer who underwent TME surgery with primary anastomosis were included, each group consisting of 32 patients. Postoperative T2-weighted MRI of the pelvis was performed at least 6 months after TME surgery and evaluated by two radiologists independently. Residual mesorectum was defined as any residual mesorectal tissue detectable after TME. Localisation of the tissue was categorised in relation to height in the pelvis and position of the level of anastomosis.

**Results:**

Residual mesorectal tissue was detected in 3.1% of TaTME patients and of 46.9% in LapTME patients (*p* < 0.001). Multivariate analysis identified only type of surgery as a significant risk factor for leaving residual mesorectum. Other known risk factors for incomplete TME, such as body mass index (BMI) and male gender, were not significant. No relation was seen between specimen quality and prevalence of residual mesorectum.

**Conclusions:**

The completeness of mesorectal excision was significantly better with TaTME than with standard laparoscopic technique.

The understanding and treatment of rectal cancer has changed over the last 30 years. The introduction of neoadjuvant therapy and enhanced surgical techniques has improved oncological and short-term patient-related outcomes. An essential feature of these surgical developments has been understanding the importance of radical TME surgery. Heald first proposed the concept of total mesorectal excision (TME) [1, 2] and others showed that pathological circumferential involvement and incomplete mesorectal excision are predictors of local recurrence [3–6].

The concept of TME surgery was introduced in the open era. Laparoscopic rectal cancer surgery improved visualisation of the surgical field and was thought to result in a significant reduction of local recurrence. However, although the COLOR II trial demonstrated the safety of a minimally invasive approach, no oncological difference was demonstrated [7]. The technique is also seen as difficult, specifically in obese male patients [8]. Furthermore, Bondeven et al. [9] showed that residual mesorectum could be demonstrated in a large proportion of patients (36%) who should have had a complete mesorectal excision, following open as well as laparoscopic surgery, based on the height of the tumour.

The transanal total mesorectal excision (TaTME) was introduced by Lacy and Adelsdorfer [10]. It is a new concept in which the most distal and difficult part of TME surgery is performed transanally using endoscopic instruments. As TaTME starts at the most distal part of the TME plane, theoretically it will result in a complete TME specimen and improve the quality of surgery. Other groups confirmed the data of Lacy and Adelsdorfer [10] and showed safe implementation [11–15].

The aim of the present study was to determine the prevalence and localisation of residual mesorectal tissue by postoperative MRI of the pelvis and compare this between TaTME and LapTME. The results of the pelvic MRI were assessed in relation to the histopathological quality of the surgical specimen.

## Methods

### Patient selection

All patients needed to provide informed consent to participate in this study as a MRI is not standard follow-up after radical rectal cancer surgery according to the Dutch rectal cancer guidelines. To avoid confusion with postoperative changes, all patients included underwent MRI at least 6 months after surgical procedure. Ethical approval was received from the Medical Ethics Review Board of the VU Medical Centre in Amsterdam.

This longitudinal study was performed in the Gelderse Vallei Hospital, which is a large teaching hospital in the central Netherlands. All patients were evaluated by a multidisciplinary cancer board and treated with neoadjuvant therapy according to Dutch guidelines [16].

Patients with a cT1–T3 rectal cancer within 10 cm from the anal verge (measured by MRI), who underwent total mesorectal excision with curative intent and primary anastomosis, were included. Previous studies showed an expected percentage of residual mesorectum of 36% following total mesorectal excision in rectal cancer patients [9]. We expected a reduction to 7.5% residual mesorectum following TaTME patients. For this reason, a total of 64 patients were needed, with 32 in each cohort (alpha 0.05, power 0.8).

**Fig. 1:**
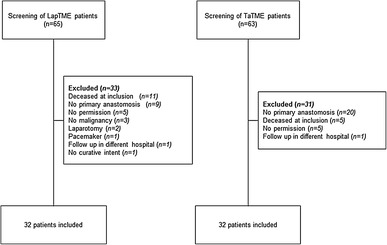
Flow-chart presenting the inclusion of patients in this study. Thirty-two patients in each group were needed (alpha 0.05, power 0.8). If patients had already undergone a postoperative MRI (> 6 months), informed consent for the use of this MRI was obtained. *LapTME* laparoscopic total mesorectal excision, *TaTME* transanal total mesorectal excision, *AV* anal verge, *MRI* magnetic resonance imaging

Between March 2012 and September 2015, 63 patients were treated with TaTME and screened for eligibility. Of these patients, 32 patients fulfilled the inclusion criteria and were willing to participate in this study. Subsequently, in order to include 32 eligible patients operated with a laparoscopic technique, 65 consecutive patients were screened from March 2012 to January 2009. Backward selection was used in order to limit the difference in operation dates, as at this time, TaTME was introduced in our hospital and the preferred treatment option. Reason for exclusion and corresponding numbers are depicted in Fig. 1.

### Surgery

Total mesorectal excision is recommended in patients with rectal carcinoma within 10 cm from the anal verge. Before 2012, standard operation was laparoscopic TME with a traditional four-trocar technique, medial to lateral as described in previous studies. Following the introduction of transanal TME (TaTME) in 2012, all patients with rectal cancer were treated by transanal approach. This technique was performed either with a one-team approach as previously described by Veltcamp Helbach et al. [12] or with a standard two-team approach as described by Arroyave et al. [17] The specimens were extracted through an umbilical incision or at an ileostomy site after placement of a wound protector. After extraction, evaluation of the denuded pelvic area and specimen was performed for persistence, respectively completeness, of residual mesorectum. The anastomosis was created using a 33-mm EEA stapler (Covidien, Mansfield, Massachusetts, USA).

### Pathology and postoperative course

The seventh edition of American Joint Committee on Rectal Cancer staging was used to describe the extent of disease progression in all patients. Quality of specimen was assessed by a specialised pathologist according to the classification provided by Nagtegaal et al. [4] Involvement of circumferential resection margins was defined as the tumour located within 1-mm distance of the resection margin. Postoperative period included all events within 30 days after index surgery. Complications were graded using the Clavien–Dindo (CD) classification, in order to separate minor complications (Grade I–II) from major complications (Grade III–V).

### Magnetic resonance imaging

To determine the amount of residual mesorectum following TME, MRI 1.5 T was used to image the pelvis. Sagittal, axial and coronal T2-weighted images of the bony pelvis were obtained, in addition to axial T1-weighted images. This is in accordance with the recommendations of the European Society of Gastrointestinal and Abdominal Radiology (ESGAR) published in 2013 [18]. Diffusion-weighted imaging (DWI) was added to better differentiate potential fibrosis, residual tumour or mesorectum.

MR images were evaluated independently by two radiologists at the Gelderse Vallei hospital. They were blinded for all clinical data, with the exception of preoperative MRI (without report). After the first evaluation, consensus reading was performed. Only when consensus was reached, patients were considered positive for residual mesorectum.

### Residual mesorectum detected on MRI

**Fig. 2:**
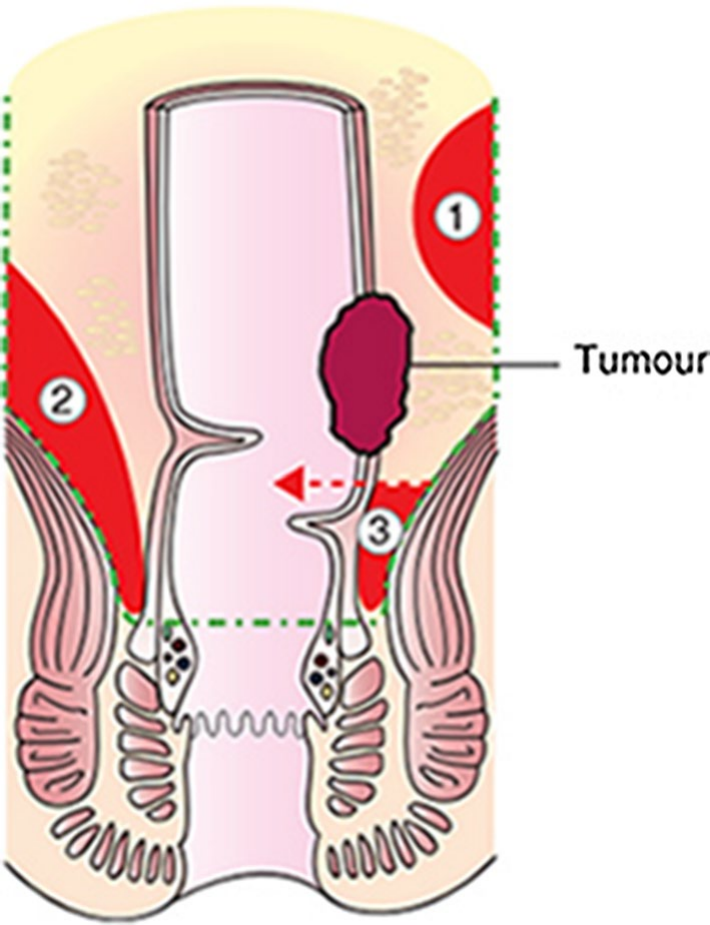
Residual mesorectum according to localisation following total mesorectal excision. Green dashed line indicates complete mesorectal excision. Red area (1) shows cranially located mesorectum independent of the distal level of resection. Red area (2) shows perianastomotic residual mesorectum in direct relation to the anastomosis. Red area (3) shows residual mesorectal tissue below the distal level of resection (red dashed line). (Reproduced with permission from Bondeven et al. [9]) © 2013 British Journal of Surgery Society Ltd Published by John Wiley & Sons Ltd. (Color figure online)

Residual mesorectum was defined as any residual mesorectal tissue detectable after total mesorectal excision. Mesorectal fatty tissue with a discernible tissue interface of fibrosis, which separates the mesorectum from the mesocolon, was considered a sign of residual mesorectal tissue [9]. The localisation of residual mesorectum was categorised in relation to height in the pelvis and position of the level of resection as described in Bondeven et al. [9] (Fig. 2).

### Oncological results

All patients received follow-up according to the Dutch rectal cancer care guidelines [16], including surveillance by CEA, colonoscopy, and imaging of liver and lungs.

### Statistical analysis

Statistical analysis was performed using SPSS version 22 for Windows and Mac (SPSS, Chicago, Illinois, USA). A *p* value ≤ 0.05 was considered statistically significant. For analysis of patient characteristics, Chi-square test and Students *t* test (Fisher–Freeman–Halton test and Mann–Whitney *U* test if not applicable) were used. Univariate and multivariate analyses were performed by logistic regression analysis.

## Results

### Baseline characteristics

Baseline characteristics were comparable between the two groups except for tumour height and tumour stage. More T3 tumours were present in TaTME group. No differences were seen in use of neoadjuvant therapy (Table 1).

**Table 1:** Demographic and clinical data

	LapTME (*n* = 32)	TaTME (*n* = 32)	*p* value
Age^a^	62.2 (59.1–65.3)	65.7 (62.4–69.1)	0.118
Sex			
Male	20 (62.5)	22 (68.8)	0.599
Female	12 (37.5)	10 (31.3)	
BMI^a^	26.0 (25.1–26.9)	27.1 (25.4–28.8)	0.263
ASA classification			
I	16 (50)	11 (34.4)	0.471*
II	15 (46.9)	19 (59.4)	
III	1 (3.1)	2 (6.3)	
History of abdominal surgery	5 (15.6)	6 (18.8)	0.740
Tumour height from AV (cm)^a^	8.7 (8.3–9.2)	7.4 (6.7–8.2)	**0.004**
Clinical T stage on MRI			
cT1	3 (9.4)	1 (3.1)	0.020*
cT2	17 (53.1)	8 (25.0)	
cT3	12 (37.5)	23 (65.7)	
Neoadjuvant therapy			
None	7 (21.9)	10 (31.3)	0.502*
RT	22 (68.8)	17 (53.1)	
CRT	3 (9.4)	5 (15.6)	
Operative time (min)^a^	164 (150–179)	206 (188–223)	**< 0.001**
Length of stay (days) (median, range)	11 (4–82)	7 (3–17)	0.074*
Postoperative complications (CD)			
Minor (I–II)	24 (75)	27 (84.4)	0.869
Major (III–V)	8 (25)	5 (15.6)	
Anastomosis height (cm)^a^	7.3 (6.8–7.8)	4.7 (4.1–5.3)	**< 0.001**
Pathology stage			
T0	4 (9.4)	2 (6.3)	0.610*
T1	2 (6.3)	5 (15.6)	
T2	11 (34.4)	12 (37.5)	
T3	14 (43.8)	13 (40.6)	
T4	1 (3.1)	0 (0%)	
Lymphnodes^a^	14.2 (11.6–16.7)	15.8 (14.0–17.7)	0.291
Completeness specimen^b^			
Complete	30 (93.8)	32 (100)	0.492*
Nearly complete	2 (3.1)	0 (0)	
Incomplete	0 (0)	0 (0)	
CRM involvement			
No	31 (96.9)	32 (100)	1.000*
Yes	1 (3.1)	0 (0)	

Statistically significant values (*p* ≤ 0.05) are given in bold

Values in parentheses are percentages or 95% confidence intervals if not mentioned otherwise

*LapTME* laparoscopic total mesorectal excision, *TaTME* transanal total mesorectal excision, *MRI* magnetic resonance imaging, *BMI* body mass index (kg/m^2^), *ASA* American Society of Anesthesiologists, *AV* anal verge, *CD* Clavien Dindo, *RT* radiotherapy, *CRT* chemoradiotherapy, *CRM* circumferential resection margin

*Calculated by Fisher–Freeman–Halton test instead of Chi-square test or Mann–Whitney *U* test instead of Student’s *t* test

^a^Values are in mean

^b^According to Quirke’s classification

### Perioperative outcomes

Operative time differed significantly between the two procedures, with a mean of 164 and 206 min for LapTME and TaTME, respectively (*p* < 0.001). In the TaTME group, no conversion during the transanal phase was necessary. In two TaTME patients, the laparoscopic part of surgery was converted to a small laparotomy because of difficulties mobilising the splenic flexure due to adhesions and to verify a serosa defect. In the laparoscopic group, two conversions occurred due to adhesions and difficulties related to large tumour size. Postoperative complications according to Clavien–Dindo classification did not differ between the two groups with major complications in 5 patients (15.6%) and 8 patients (25%) in TaTME and LapTME patients (*p* = 0.226). Median length of stay was the same after TaTME and LapTME (*p* = 0.869) (Table 1), respectively.

### Residual mesorectum

After first evaluation of magnetic resonance images, agreement was found in 59.4% of cases. After this first evaluation, consensus reading occurred and consensus was obtained in all cases. MRI-detected residual mesorectum was identified in one patient (3.1%) after TaTME, and in 15 patients (46.9%) after laparoscopic LapTME (*p* < 0.001).

Univariate analysis demonstrated that tumour height and type of surgery differed significantly in terms of residual mesorectum with *p* values of < 0.001 and 0.008, respectively (Table 2). Multivariate analysis of these two factors identified only type of surgery as a statistically significant risk factor for residual mesorectum with an odds ratio of 0.048 (95% CI 0.006–0.406, *p* value 0.005) (Table 3). Subanalysis within the laparoscopic group showed no significant differences in tumour height, BMI or gender concerning presence of residual mesorectum on MRI.

**Table 2:** Magnetic resonance imaging-detected residual mesorectum

	No. of patients (*n* = 64)	Residual mesorectum (*n* = 16)	No residual mesorectum (*n* = 48)	*p* value
Type of surgery				
TaTME	32	1 (3.1)	31 (96.9)	**< 0.001**
LapTME	32	15 (46.9)	17 (53.1)	
Sex				
Male	42	10 (23.8)	32 (76.2)	0.761
Female	22	6 (27.3)	16 (72.7)	
BMI^a^		27.5	26.2	0.233
		(25.6–29.4)	(25.1–27.3)	
ASA				
I	27	6 (22.2)	21 (77.8)	0.628*
II	34	10 (29.4)	24 (70.6)	
III	3	0 (0.0)	3 (100.0)	0.100
History of abdominal surgery				
Yes	11	2 (18.2)	9 (81.8)	0.716*
No	53	14 (26.4)	39 (73.6)	
Tumour distance from AV (cm)^a^		9.1	7.7	**0.008**
		(8.6–9.6)	(7.1–8.3)	
Neoadjuvant therapy				
None	17	4 (23.5)	13 (76.5)	0.838*
RT	39	11 (28.2)	28 (71.8)	
CRT	8	1 (12.5)	7 (87.5)	
Pathology T stage				
pT0	6	3 (50.0)	3 (50.0)	0.224*
pT1	7	1 (14.3)	6 (85.7)	
pT2	23	3 (13.0)	20 (87.0)	
pT3	27	9 (33.3)	18 (66.7)	
pT4	1	0 (0.0)	1 (100.0)	
Completeness specimen^b^				
Complete	62	14 (22.6)	48 (77.4)	0.060*
Nearly complete	2	2 (100.0)	0 (0.0)	
Incomplete	0	0 (0.0)	0 (0.0)	
CRM involvement				
No	63	16 (25.4)	47 (74.6)	1.000*
Yes	1	0 (0.0)	1 (100.0)	

Statistically significant values (*p* ≤ 0.05) are given in bold

Values in parentheses are percentages or 95% confidence intervals

*TaTME* transanal total mesorectal excision, *LapTME* laparoscopic total mesorectal excision, *BMI* body mass index (kg/m^2^), *ASA* American Society of Anesthesiologists, *AV* anal verge, *CRM* circumferential resection margin

*Calculated by Fisher–Freeman–Halton test instead of Chi-square test

^a^Values are in mean

^b^According to Quirke’s classification

**Table 3:** Multivariate analysis of risk factors for presence of residual mesorectum

Factor	Adjusted odds ratio	95% confidence interval	*p* value
Type of surgery			
TaTME (*n* = 1)	0.048	0.006–0.406	**0.005**
LapTME (*n* = 15)			
Increase of 1 cm in tumour height from AV	1.68^a^	0.943–2.98	0.078

Statistically significant value (*p* ≤ 0.05) is given in bold

*TaTME* transanal total mesorectal excision, *LapTME* laparoscopic total mesorectal excision, *AV* anal verge

^a^Measured for continuous variable

**Fig. 3:**
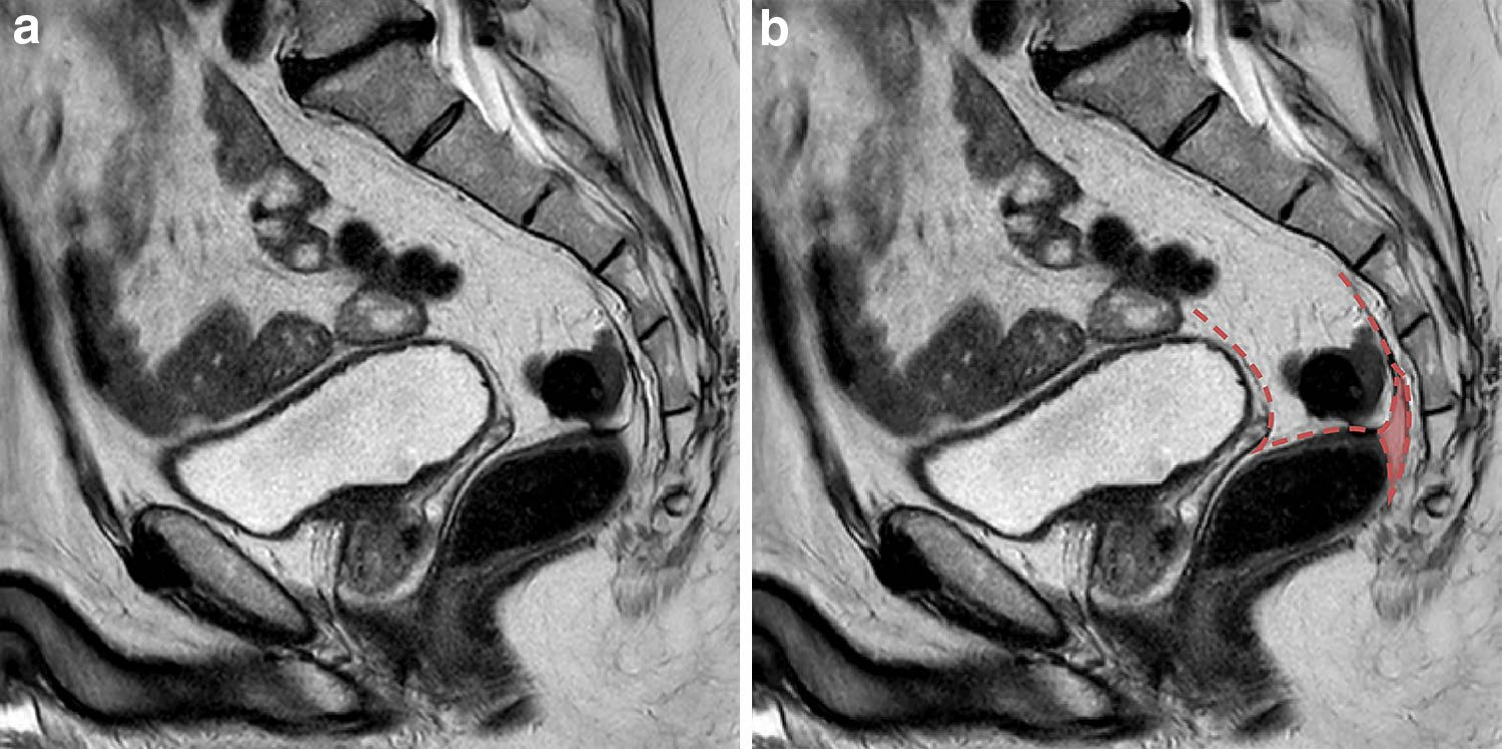
MRI-detected residual mesorectum following laparoscopic total mesorectal excision (LapTME). **a, b** Sagittal T2-weighted MR-images showing residual mesorectum below the anastomosis and perianastomotic. **b** Residual mesorectum coloured in red. Red dashed line is showing the level of dissection and anastomosis after total mesorectal excision. (Color figure online)

**Fig. 4:**
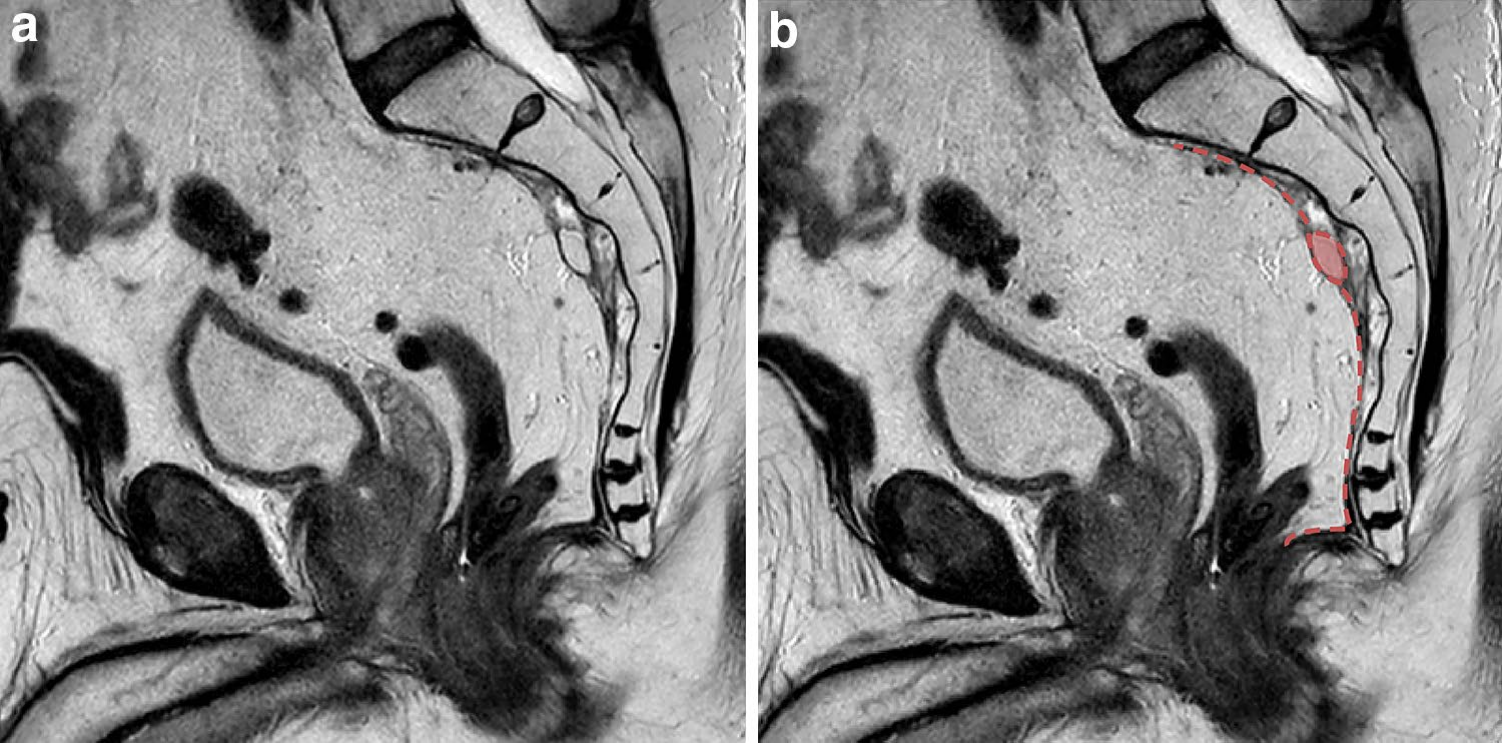
MRI-detected residual mesorectum following transanal total mesorectal excision (TaTME). **a, b** Sagittal T2-weighted MR-images showing cranially located residual mesorectum in relation to the anastomosis. **b** Residual mesorectum coloured in red. Red dashed line showing the level of dissection and anastomosis after TaTME. (Color figure online)

The localisation of the residual mesorectal tissue in the LapTME group was below the distal level of resection in 9 patients (60%) and perianastomotic in 6 patients (40%) (Fig. 3). The residual mesorectum found in the one patient following TaTME was cranially located independent of the distal level of resection (Fig. 4).

### Pathology and magnetic resonance imaging

Pathology reports showed no differences in pathology T stage of the tumour. One patient in the TaTME group was graded as pT4 because of growth into the uterus, which was already suspected during surgery. Only in this patient, a positive circumferential resection margin (3.1%) was reported.

No relation was seen between specimen quality and prevalence of residual mesorectum. All specimens were graded as a complete mesorectum, with the exception of two patients in the LapTME group who were reported as nearly complete (6.3%). In these latter patients, residual mesorectum was identified on MRI and located perianastomotic and below the distal resection level. Pathology findings of the remaining 14 patients with MRI-detected residual mesorectum were reported as complete specimens.

### Recurrences

No local recurrence was found in any of the included patients, with a minimum oncological follow-up of 13.8 months (range 13.8–96.5). Three patients, two LapTME and one TaTME patient, had systemic recurrence; one diagnosed with pulmonary, one with hepatic and one with pulmonary, hepatic and lymphatic metastases. In all three patients, residual mesorectum was not identified on postoperative MRI.

## Discussion

This study reports the first postoperative MRI data on completeness of mesorectal excision after both laparoscopic and transanal TME. It demonstrates a significant difference in the completeness of the mesorectal excision in favour of TaTME when standard laparoscopic technique is compared with a combined transanal and laparoscopic approach. After multivariate analysis, only type of surgery was a significant risk factor for residual mesorectum, whereas factors traditionally viewed as contributing to poor pathological results, such as BMI, male sex and tumour height, were not significant. In 14 patients (22.6%), residual mesorectum was found on postoperative MRI, while their specimen was assessed as complete by the pathologist.

In the current series, 47% of patients operated with a laparoscopic technique showed residual mesorectum. While we were surprised to find such a high percentage, these data are supported by findings of Bondeven et al. [9]. These authors reported residual mesorectum in 36% of patients who, based on the height of the tumour, should have had a total mesorectal excision. However, the majority of these patients underwent open TME. Despite this difference, the most prevalent types of residual mesorectum we found were below the anastomosis and perianastomotic, similar to the Bondeven’s study [9]. This suggests that the mesorectum is dissected inward during the dissection of the distal rectum, leaving mesorectum behind.

TaTME is a new and promising technique for the treatment of rectal cancer. In TaTME, the most distal and difficult part of TME surgery is performed transanally with endoscopic instruments. As TaTME starts at the most distal part of the TME plane, theoretically it will result in a complete TME specimen and improve the quality of surgery. The current data show that TaTME achieves a significantly lower percentage of residual mesorectum, with residue observed in only one patient. The present study also evaluated the impact of the type of procedure on the height of the anastomosis. TaTME patients had a significantly lower anastomosis. Although it is not surprising that more extensive mesorectal excision results in a lowering of the anastomosis, we do not yet know what impact this will have. While it could well influence functional results, the only data currently available suggest that TaTME will have little impact on long-term functional results [19].

The pathological specimen is often thought to be an important predictor of the quality of surgery and to predict the prognosis. The relevance of a complete resection of the total mesorectum is underlined by data from the Dutch TME trial showing that incomplete specimen was associated with an increased risk for local and distant recurrence [4]. By contrast, the present study did not show an association between the specimen quality evaluated by pathological examination and residual mesorectum on MRI as shown in Table 2. Simply said, the quality of the pathological specimen does not seem to reflect the completeness of mesorectal excision. Although the grading system classified by Quirke [4] defines very precisely the exact plane of surgery, the distal resection, which is the most difficult part of the surgery, is less defined and therefore possibly mistaken by the pathologist. When the mesorectum is stapled off it is difficult to distinguish complete and partial mesorectal excision by pathology.

Bondeven et al. [9] also showed this discrepancy between pathological results and the amount of residual mesorectum on MRI. Quality assessment of the specimen, when reassessed by the pathologist on standardised photographic documentation, changed in 42% of cases, again suggesting that the pathologists are not able to judge whether a specimen is complete or whether residual mesorectum is left behind below the anastomosis. In the current series, it was not possible to re-evaluate the quality of the specimens, as the older specimens were not routinely photographed. In a previous study, we reported a significant difference in the quality of the specimen between patients operated laparoscopically and with TaTME [20]. The quality of the specimens in the current series was consistently good, so the difference between the two techniques could not be confirmed.

Of course, the clinical relevance of MRI-detected residual mesorectum remains a point of discussion. Recurrence rates have decreased significantly over the past years, with a combination of improved surgical technique, improved preoperative staging and neoadjuvant therapy resulting in a local recurrence rate of 5% after both open and laparoscopic surgeries [7]. Nonetheless, the morbidity and mortality rates associated with a local recurrence remain substantial. Furthermore, Syk et al. [21] reported evidence of residual mesorectum and the location of recurrences. Residual mesorectum was found on MRI in 50 of the 99 patients with a local recurrence, while the site of the recurrence was the lower pelvis in the majority of the patients [20].

Although all patients had a minimal follow-up of at least 1 year, no local recurrences were seen. This could be due to selection bias since patients with local recurrence were possibly not willing or able to participate. We previously reported two local recurrences in the first 80 patients. Few series have described local recurrence after TaTME and the most recent update of literature, with an overall follow-up of 18.9 months, reported a local recurrence rate of 4%. The same review showed an involvement of the CRM in 4.7% [22]. In the COLOR II trial [7], a higher percentage of recurrence after laparoscopic approach compared to open surgery was seen in patients with mid-rectal cancer. Pathological analysis, however, showed no difference in quality of the specimen or in resection margin. As stated before, ‘coning in’ during dissection, resulting in residual mesorectum for patients who need total mesorectal excision, could explain the difference found in oncological outcome.

While it is clear from our results that TaTME significantly reduces the percentage of patients with residual mesorectum, one could debate the use of MRI in the evaluation of residual mesorectum since this is not yet validated. After the first evaluation, there was agreement of the radiologists in 59.4% of cases. After consensus reading, consensus was reached in all patients as the radiologists became more familiar with identifying residual mesorectum on MRI which implicates a learning curve. We only considered patients positive for residual mesorectum when both radiologists agreed on the presence of residual mesorectum on MRI. Moreover, we included patients with MRI at least 6 months after surgery to avoid confusion with postoperative changes. We are aware that detection of residual mesorectum on postoperative MRI is not yet validated and that results should be interpret with caution.

The current findings add to existing evidence that TaTME is a safe and effective technique for the treatment of mid-rectal and lower rectal cancer. The international registry recently reported on the first 720 cases and showed acceptable short-term patient outcomes [15]. Data from the most experienced centre even suggest short-term benefits [23]. However, before TaTME is accepted as a standard for rectal cancer surgery, more long-term data are needed and it must be proven that it is as safe as other existing techniques. We have therefore initiated the COLOR III trial [24], a randomised controlled trial comparing TaTME with laparoscopic surgery for rectal cancer.

In conclusion, residual mesorectum on postoperative magnetic resonance imaging is found more frequently after laparoscopic surgery in comparison to transanal surgery for patients with mid- and low-rectal cancer. Further studies are needed to evaluate long-term oncological outcome and to validate our results.
